# Diagnostic evaluation of long-read nanopore sequencing for pediatric CNS tumors

**DOI:** 10.1186/s40478-026-02466-w

**Published:** 2026-10-27

**Authors:** Sandra Ferreyra Vega, Ryan Shun-Yuen Kwan, Ida Rahmqvist, Elizabeth Schepke, Magnus Sabel, Thomas Olsson Bontell, Teresia Kling, Helena Carén

**Affiliations:** 1 Sahlgrenska Center for Cancer Research, Department of Medical Biochemistry and Cell biology, Institute of Biomedicine, Sahlgrenska Academy, University of Gothenburg, Medicinaregatan 1F, 405 30 Gothenburg, Sweden; 2 Department of Pediatrics, Institute of Clinical Sciences, Sahlgrenska Academy, University of Gothenburg, Gothenburg, Sweden; 3 Childhood Cancer Centre, Queen Silvia Children’s Hospital, Sahlgrenska University Hospital, Gothenburg, Sweden; 4 Department of Physiology, Institute of Neuroscience and Physiology, Sahlgrenska Academy, University of Gothenburg, Gothenburg, Sweden; 5 Department of Clinical Pathology, Sahlgrenska University Hospital, Gothenburg, Sweden

**Keywords:** Pediatric CNS tumors, Long-read nanopore sequencing, 2021 WHO CNS classification, Methylation profiling, Tumor diagnostics, Childhood cancer

## Abstract

Long-read nanopore sequencing enables methylation-based CNS tumor classification while providing additional genomic information within a single assay. However, its performance across molecular features relevant to pediatric CNS tumor diagnostics and across available methylation classifiers remains incompletely characterized. We evaluated nanopore sequencing for integrated molecular characterization of pediatric CNS tumors and compared with data from DNA methylation arrays and Illumina short-read sequencing used in the clinical diagnostic work-up. Twenty tumor samples representing three major pediatric CNS tumor groups (gliomas, ependymomas and medulloblastomas) were subjected to long-read nanopore sequencing, which generated robust long-read data across all tumor groups, with a mean genome coverage of approximately 40x. Nanopore-derived copy-number profiles showed good concordance with methylation array-derived profiles (median Pearson *r* = 0.816). Nanopore evidence was identified for 74/78 (95%) clinically relevant small variants detected by Illumina sequencing, although 53/78 (68%) were recovered as exact high-confidence nanopore calls. Structural variant profiles showed limited breakpoint overlap and differed in the number, type, and size distribution of detected events between Illumina and Nanopore sequencing. Nanopore-derived methylation classifications were largely concordant with integrated WHO CNS5 diagnoses. Ependymomas, medulloblastomas, and pilocytic astrocytomas, were concordantly classified across all evaluated classifiers, although not all classifications reached the respective classifier-specific confidence thresholds. In contrast, diffuse gliomas showed greater variability in classification across classifiers. Downsampling indicated that concordance with integrated WHO CNS5 diagnoses remained high at substantially reduced sequencing depths, whereas the proportion of classifications reaching classifier-specific confidence thresholds was more classifier dependent. Long-read nanopore sequencing enables integrated molecular characterization of pediatric CNS tumors within a single assay, although concordance with established methods varied by genomic feature and tumor type. The preservation of methylation classification performance at reduced sequencing depth supports the feasibility of shorter sequencing workflows. Prospective studies are required to establish performance and turnaround time in routine clinical diagnostics.

**Supplementary Information:**

The online version contains supplementary material available at https://doi.org/10.1186/s40478-026-02466-w.

## Introduction

Pediatric central nervous system (CNS) tumors are the most common solid malignancies in children and remain a leading cause of cancer-related morbidity and mortality [14]. Unlike adult CNS tumors, pediatric CNS tumors arise in the context of distinct developmental and biological processes and are characterized by unique molecular drivers, epigenetic landscapes, and clinical behaviors. As a result, diagnostic frameworks developed for adult CNS tumors are often not directly applicable to pediatric cases, underscoring the need for age-specific classification and diagnostic approaches. Accurate tumor diagnosis is therefore essential in children, as it directly influences risk stratification, treatment strategies, and long-term outcomes.

The importance of molecular diagnostics in CNS tumor classification has been further emphasized in the 2021 World Health Organization (WHO) Classification of CNS tumors, which adopts an integrated diagnostic approach combining histopathology with molecular and epigenetic features [22]. This paradigm shift is especially relevant in pediatric neuro-oncology, where tumors with similar histological appearances may represent biologically distinct entities with markedly different prognoses and therapeutic responses.

DNA methylation profiling has emerged as a robust and highly informative method for the classification of pediatric CNS tumors, providing objective and highly reproducible tumor stratification and have contributed to the identification of novel pediatric-specific tumor entities [1, 15]. The Infinium MethylationEPIC (EPIC) array is widely regarded as the current gold standard technique in clinical neuro-oncology. However, array-based methylation profiling presents several practical limitations that can restrict its utility in routine clinical diagnostics, including relatively high costs, long turnaround times, and the need to multiplex samples for processing. These limitations have prompted increasing interest in sequencing-based approaches, such as Oxford Nanopore Technologies (ONT) long-read sequencing, which offers the potential to integrate methylation profiling with comprehensive genomic characterization within a single assay. Further, long-read nanopore sequencing has been shown to provide real-time data acquisition, raising the possibility of intra-operative rapid diagnostic classification of CNS tumors [4, 19].

In this study, we evaluated the clinical utility of long-read nanopore sequencing for molecular characterization of pediatric CNS tumors according to the fifth edition of the WHO CNS tumor classification criteria (WHO CNS5) published in 2021. We compared nanopore-derived copy-number variation (CNV), single-nucleotide variants, structural variants, and methylation profiles with corresponding data from next-generation short-read sequencing and methylation arrays. We further evaluated the performance of different nanopore-based methylation classifiers for diagnostic classification of these tumors.

## Materials and methods

### Study cohort

A clinical consecutive cohort of pediatric patients diagnosed with primary CNS tumors during 2013–2025 in the Västra Götaland region in Sweden was evaluated for inclusion. Signed informed consent was obtained from the guardians of the children undergoing CNS tumor resection at the Sahlgrenska University Hospital (Dnr: 604 − 12, Regional Ethical Review Board in Gothenburg, Sweden). Tumor tissue samples were snap frozen in liquid nitrogen within 1–20 min following surgical resection and subsequently long-term stored at -140°C until DNA extraction. Patients with fresh-frozen (FF) tumor samples available for long-read nanopore sequencing and corresponding Illumina short-read sequencing and methylation array data generated as part of the routine clinical diagnostics [2] were included for further analysis. We selected patients diagnosed with three major pediatric CNS tumor groups including gliomas (diffuse gliomas and pilocytic astrocytomas), ependymomas and medulloblastomas. To provide a uniform diagnostic reference across the cohort, all cases were evaluated according to WHO CNS5. Cases originally diagnosed according to WHO CNS5 retained their diagnosis, whereas cases diagnosed according to earlier WHO CNS classifications were retrospectively reviewed and reclassified according to WHO CNS5 based on the available histopathological and molecular findings. Diagnostic assessment included conventional histopathology and immunohistochemistry, complemented by tumor type-specific molecular analyses, including fluorescence in-situ hybridization (FISH), next generation sequencing (NGS)-based mutation analysis, NGS-based RNA sequencing and DNA methylation profiling, as appropriate for the respective tumor type. The combination of diagnostic modalities varied according to tumor type and the diagnostic work-up performed at the time.

### Long-read nanopore sequencing

#### Library preparation and sequencing

Extraction of genomic DNA (gDNA) from approximately 10 mg of FF tumor tissue was carried out with the DNeasy Blood and Tissue Kit and TissueLyser (Qiagen) according to the manufacturer’s instruction, followed by fragmentation of 2 ug gDNA with Covaris^®^ g-TUBES to get an average gDNA target size of 10 kb. The resulting DNA fragment length was verified using the TapeStation Screen Tape Assay 4150 (Agilent) before and after shearing gDNA. DNA sequencing libraries were prepared with 1 ug sheared gDNA using the NEBNext Companion module (New England Biolabs) and Ligation Sequencing Kit (SQK-LSK114, ONT long-read nanopore sequencing), according to the developer’s protocol. Each library was sequenced individually on a flow cell (FLO-PRO114M) for 72 h on a PromethION^TM^ 2 (P2) Solo instrument. The MinKNOW software was employed to initiate and monitor the sequencing runs.

### Data processing

#### Nanopore basecalling, modified-base detection and alignment

Sequencing reads (POD5 files) were basecalled post-sequencing with Dorado base caller (v1.1.1) using the super accurate mode and 5hmC_5mC methylation calling in all CG context (dna_r10.4.1_e8.2_400bps_sup@v5.2.0) and mapped to the Genome Reference Consortium Human Build 38 patch release 14 (GRCh38.p14) during basecalling. Reads with a basecalling quality score below 10 were excluded.

#### Tumor-only genomic analysis using Lumos

Tumor-only genomic analysis was performed using the Lumos workflow implemented in Nextflow v25.10.0. Input BAM files were restricted to reads mapped to standard chromosomes (chr1–chr22, chrX, and chrY), followed by sorting, merging, and indexing using SAMtools v1.20.

Small germline variants used for haplotype phasing were called using Clair3 v1.0.11, with the Clair3 model selected according to the nanopore basecalling model detected from the input BAM files. Clair3 variants were phased using LongPhase v1.7.3. Phase blocks were subsequently processed using the hapcorrect module of Wakhan v0.4.0 (doi: https://doi.org/10.64898/2025.12.11.25342098) to obtain a rephased variant set. Sequencing reads were assigned to the resulting haplotypes using WhatsHap v2.3, producing haplotagged BAM files for downstream allele-specific analyses.

Structural variants (SVs) were detected from haplotagged reads using Severus v1.6. SV analysis incorporated a GRCh38 VNTR annotation and a 1000 Genomes-derived panel of normals to support filtering of recurrent variants. Tumor-only somatic single nucleotide variants (SNVs) and small indels were called independently from the merged tumor BAM using DeepSomatic v1.9.0.

Workflow processes were executed on the UPPMAX high-performance computing environment using the SLURM workload manager, with software dependencies provided through environment modules or Singularity containers as defined by the Lumos workflow. Software versions and key analysis parameters are summarized in Supplementary Material1.

### Copy-number variation analysis

Copy-number alterations, tumor purity, ploidy, allele-specific copy-number states, and loss of heterozygosity (LOH) were analyzed using Wakhan v0.4.0, integrating aligned sequencing reads, phased variants, haplotype information, and SVs. Copy-number analysis was performed using a genomic bin size of 50 kb, corresponding to the default bin size implemented in the Wakhan workflow. COSMIC cancer-gene annotations were included in the analysis. The highest-ranked purity/ploidy solution reported by Wakhan was used for downstream analyses.

Total CNV profiles were generated by combining the inferred copy-number states of both haplotypes. Allele-specific CNV profiles displaying individual haplotype copy-number states and phased coverage were generated to visualize haplotype-specific gains, losses, and LOH.

The 50-kb genomic bin size defines the nominal resolution of the read-depth and B-allele-frequency-based analysis. Segmentation additionally incorporates somatic SV breakpoints identified by Severus, enabling breakpoint-guided segmentation and haplotype-specific copy-number assignment. Haplotype-specific CNV resolution is dependent on the extent of informative phasing; alterations within unphased or poorly phased regions may therefore be detectable at the total-copy-number level without reliable assignment to an individual haplotype.

#### Cross-platform CNV concordance analysis

CNV concordance between matched nanopore and methylation array data (EPIC v1) was evaluated. CNV profiles retrieved from the array data were generated in GRCh38/hg38, using the conumee2 R package (http://github.com/hovestadtlab/conumee2) with standard parameters, and analyses were restricted to autosomes. For nanopore sequencing, haplotype-specific segment coverage reported by Wakhan was summed across HP1 and HP2 and normalized to the sample-specific length-weighted median autosomal coverage, followed by log2 transformation to obtain a relative coverage-derived CNV signal. The continuous coverage signal, rather than Wakhan-inferred integer copy-number states, was used for this comparison.

To account for differences in genomic resolution between the platforms (nanopore sequencing vs. methylation array (EPIC v1)), the nanopore relative CNV signal was evaluated at the hg38 coordinates of EPIC v1 probes. For each array CNV segment, the median nanopore signal across EPIC probe positions within that segment was calculated and compared with the corresponding EPIC segment mean. Cross-platform pattern concordance was quantified using Pearson correlation. Spearman's rank correlation and cosine similarity were additionally calculated as complementary measures of profile similarity. Numerical agreement was additionally assessed using the mean absolute difference between the paired methylation array and nanopore relative CNV signals.

As a specificity analysis, each CNV profile retrieved from methylation array data was compared with the corresponding CNVs from nanopore data of all 20 tumors. For each tumor, the matched correlation was compared with the median of the 19 mismatched correlations using a paired one-sided Wilcoxon signed-rank test. The rank of the matched nanopore profile among all 20 nanopore profiles was also determined. To assess whether correlation-based concordance was influenced by the extent of CNVs within individual profiles, CNV signal variability was quantified using the standard deviation (SD), interquartile range (IQR), and range of the segment-level relative CNV signals for both platforms. Associations between CNV signal variability and matched methylation array–nanopore Pearson correlation coefficients were assessed using Spearman's rank correlation. P-values from the six variability measures tested were adjusted using the Benjamini–Hochberg false discovery rate (FDR) procedure (Supplementary Material 2).

### Target-gene variant analysis

Illumina short-read variants were called from matched tumor–healthy normal sequencing data using Mutect2 (GATK v4.1.7.0), Strelka (v2.9.10) and FreeBayes (v1.3.2). Mutect2 used the 1000 Genomes panel of normals and gnomAD v2.1.1 as a germline resource, while FreeBayes was run with a minimum alternate allele fraction of 0.03 and a minimum alternate read count of 2. Variants were annotated using SnpEff (v4.3t) and VEP (v99). Software versions and key analysis parameters are summarized in Supplementary Material 1.

Variant calls from Illumina short-read sequencing and nanopore long-read sequencing were converted into harmonized tables containing genomic coordinates, alleles, variant type, variant allele frequency, sequencing depth and functional annotation. Illumina consensus variants were defined as variants detected by at least two of Mutect2, Strelka and FreeBayes. When FreeBayes results were unavailable, concordant calls from both Mutect2 and Strelka were required. Concordance required an exact match of chromosome, position, reference and alternate allele.

Target-gene analyses were restricted to a predefined panel of clinically relevant genes [6]. Target-gene membership was assigned using genomic coordinates rather than gene symbols. Variants were retained if they were annotated as missense, frameshift, in-frame insertion/deletion, stop-gained/stop-lost, splice-site or structural-interaction variants, or classified as Pathogenic/Likely Pathogenic in ClinVar [10].

Target variants were compared using a stepwise evidence framework. Illumina consensus variants were first compared with high-confidence nanopore calls (Lumos PASS) using exact variant matching based on genomic position and allele change. Variants without an exact match were subsequently evaluated in the complete Lumos callset, the Human Variation Workflow from ONT, and manual inspection of nanopore read alignments in the Integrative Genomics Viewer (IGV, v2.19.7) when no computational support was identified. The Human Variation Workflow was used only as a secondary source of supporting evidence and not for primary nanopore variant calling. Small variants in this workflow were called using Clair3 v1.0.8, and the workflow configuration used here did not apply matched-normal or panel-of-normals filtering to these calls. Additional computational and manual evidence was retained separately from primary Lumos PASS detection. Conversely, high-confidence nanopore variants absent from the Illumina consensus were queried against the individual Illumina caller outputs (Mutect2, Strelka and FreeBayes) to identify exact lower-confidence calls or alternative variant representations at the same or nearby genomic position. Gene-level cross-platform evidence was summarized using an oncoprint, in which a sample–gene combination was considered supported by both technologies if at least one individual target variant showed evidence in both platforms. Primary calls and additional computational or manual evidence were combined in this gene-level summary but retained as separate evidence levels in the variant-level analysis.

To assess the effect of variant allele frequency (VAF) on nanopore variant recovery, all Illumina consensus SNVs were additionally evaluated irrespective of target-gene membership. Nanopore recovery was calculated across rolling Illumina VAF windows (± 0.05 VAF) using exact variant matches. The association between median Illumina SNV VAF and nanopore SNV recovery across samples was assessed using Spearman's rank correlation. Associations between sample-level SNV recovery and tumor purity estimates were assessed using Spearman's rank correlation.

### Structural variant analysis

SVs were identified from Illumina short-read sequencing using Manta v1.6.0 and from long-read nanopore sequencing using Severus v1.6. Analyses were restricted to PASS-filtered somatic calls on chromosomes (chr1–chr22, chrX and chrY).

Cross-platform SV overlap was assessed using a custom bidirectional breakpoint-based approach, in which a breakpoint was considered supported if any breakpoint in the other callset occurred on the same chromosome within ± 500 bp, irrespective of SV type. For two-breakpoint events, support for both, one, or neither breakpoint was recorded. Breakpoint tolerances of 100–1,000 bp were additionally evaluated as a sensitivity analysis. SV size distributions and size-stratified breakpoint overlap were assessed for SV classes with defined lengths.

As Illumina and nanopore sequencing were performed on spatially distinct tumor samples, the analysis reflects combined cross-sample and cross-platform overlap and cannot distinguish technical differences from spatial intratumor heterogeneity.

### Methylation-based classification

#### Nanopore methylation preprocessing

Nanopore methylation data was processed to generate classifier-specific inputs according to the feature requirements of each classification method. Methylation calls from phased and ungrouped bedMethyl outputs were combined when required, and genomic positions represented in multiple files were summarized by combining valid coverage and modified-base counts. Nanopore methylation sites were mapped to the corresponding methylation array EPICv1 probe coordinates.

#### Nanopore-based CNS tumor classification

Nanopore-based diagnostic classification was performed using MNP-Flex v12.8 [13], RapidCNS^2^ [12], Sturgeon [19], NanoDx (including PanNanoDx) [9, 24] and M-PACT [17]. As the classifiers use different scoring systems, classifier-specific score and confidence categories were applied. The thresholds used for categorization in this study were: MNP-Flex, ≥ 0.30 (above cutoff); Rapid-CNS^2^, ≥ 0.70 (above cutoff); Sturgeon, ≥ 0.95 (high confidence), 0.80 –< 0.95 (confident), and < 0.80 (inconclusive); NanoDx and PanNanoDx, > 0.70 (high score), 0.30 – 0.70 (intermediate score), and < 0.30 (low score); and M-PACT, ≥ 0.70 (above cutoff). Published thresholds differ between classifier versions and implementations and should therefore not be considered directly comparable across methods.

Methylation classifiers use diverse architectures, from traditional machine learning to deep learning models designed for sparse sequencing data. MNP-Flex, which is trained on the same dataset as the Heidelberg Epignostix CNS Tumor methylation array-based classifier, applies XGBoost with binarized methylation values to achieve high accuracy across multiple platforms. Among the other nanopore-based pipelines, Rapid-CNS^2^ retrains a random forest model for each sample to adapt sparse nanopore data to array-based references, whereas NanoDx and PanNanoDx use pretrained neural networks (crossNN) for immediate predictions. Sturgeon employs a patient-agnostic neural network trained to handle extreme data missingness, while M-PACT uses a deep neural network ensemble with CpG imputation to improve classification from sparse methylomes. Full software versions, model identifiers, preprocessing settings, and analysis-defining parameters are provided in Supplementary Material 1.

### Sequencing-depth and modified-base sensitivity analyses

#### Generation of reduced-depth datasets

To evaluate the effect of sequencing depth on nanopore methylation-based classification, nested datasets representing approximately 1×, 5×, and 10× genome coverage were generated and compared with the full-depth datasets (> 30×). Reduced-depth datasets were generated sequentially from BAM files using cumulative file-size thresholds of 3.1, 15.5, and 31.0 GB for approximately 1×, 5×, and 10× coverage, respectively.

BAM files were processed using SAMtools v1.24 and HTSlib v1.24, including merging, coordinate sorting, validation using samtools quickcheck, and indexing. Before methylation analysis, alignments were restricted to canonical autosomes, and chromosomes X and Y.

#### Methylation profiling and diagnostic classification across sequencing depths

CpG methylation was quantified using Modkit v0.6.4 pileup with the GRCh38.p14 reference genome and the options cpg, modified-bases 5mC, 5hmC, and combine-strands. Separate methylation profiles were generated for the 1×, 5×, and 10× datasets. To assess the effect of modified-base representation, methylation-based classifications were evaluated using 5mC-only and combined 5mC + 5hmC profiles. Rapid-CNS^2^, NanoDx, PanNanoDx, Sturgeon, and M-PACT were evaluated across the different sequencing depths. A methylation classification was considered concordant when the standardized top-ranked classifier assignment matched the corresponding WHO CNS5 diagnosis. Classification confidence was evaluated separately using the classifier-specific score thresholds described above.

For each classifier, methylation profile, and sequencing-depth group (1×, 5×, 10×, and > 30×), diagnostic concordance and classifier confidence were evaluated separately. The proportions of concordant and discordant classifications within each classifier-specific confidence category were compared across sequencing depths and between 5mC-only and combined 5mC + 5hmC profiles. The proportion of classifications reaching the classifier-specific high-confidence threshold and the proportion concordant with the WHO CNS5 diagnosis were additionally summarized across sequencing depths for each classifier and methylation profile.

#### Turnaround-time estimation

Potential time to methylation-based classification at reduced sequencing depth was estimated from the sequencing yield observed in the full nanopore sequencing runs. Using approximately 40× genome coverage generated over 72 h as a representative sequencing rate, estimated times to reach 1×, 5×, and 10× coverage were approximately 1.8, 9, and 18 h, respectively. Downstream BAM preparation and modified-base processing times were estimated from measured processing rates, and classifier runtimes were estimated from workflow output timestamps. These estimates represent computational projections from the retrospective sequencing data.

### DNA methylation array profiling and analysis

Methylation array (EPIC v1) data were obtained from The Swedish Childhood Tumor Biobank [2], generated from FF tumor tissue, or generated for this study when data was unavailable from the childhood tumor biobank as previously described [8]. Raw methylation array data (IDAT files) were processed with the statistical software R version 4.4.1 [18] with the R-package minfi. Epignostix classifier version 12.8 [7, 16] was employed for tumor methylation subclassification (subclass score ≥ 0.90).

## Results

### Description of study cohort

A total of 20 CNS tumor tissue samples from 20 children diagnosed according to the WHO CNS5 were included in the study. The cohort comprised eight gliomas (IDH-mutant astrocytoma grade 4 (*n* = 1), diffuse pediatric-type high-grade glioma, H3-wildtype and IDH-wildtype grade 4 (*n* = 1), IDH-wildtype glioblastoma grade 4 (*n* = 2) and pilocytic astrocytoma grade I (*n* = 4)), five ependymomas (posterior fossa group A grade 3 (*n* = 2), posterior fossa group B grade 2 (*n* = 1), supratentorial ZFTA-fusion-positive grade 3 (*n* = 1) and spinal myxopapillary grade 2 (*n* = 1)), seven medulloblastomas (non-WNT/non-SHH group 3 grade 4 (*n* = 1), non-WNT/non-SHH group 4 grade 4 (*n* = 3), WNT activated grade 4 (*n* = 2) and SHH-activated and TP53-wildtype grade 4 (*n* = 1)). Two of the glioma patients were diagnosed with Constitutional Mismatch Repair Deficiency (CMMRD) (GU-pBT-178 and GU-pBT-31). For each tumor sample, matched Illumina short-read sequencing and DNA methylation array data were available, enabling direct comparison of nanopore-based molecular analyses with established clinical reference methods. The clinical characteristics of the patients are shown in Table 1.

**Table 1:** Clinical information of patients included in the study cohort

Variables	*n* = 20
Mean age at diagnosis (years)	8.4 (1.9–17 years)
Gender	
Female	9
Ratio Female/male	1:1.2
Tumor diagnosis (WHO CNS5)	
Glioma	8
Ependymoma	5
Medulloblastoma	7

### Long-read nanopore sequencing performance across pediatric CNS tumors

**Fig. 1:**
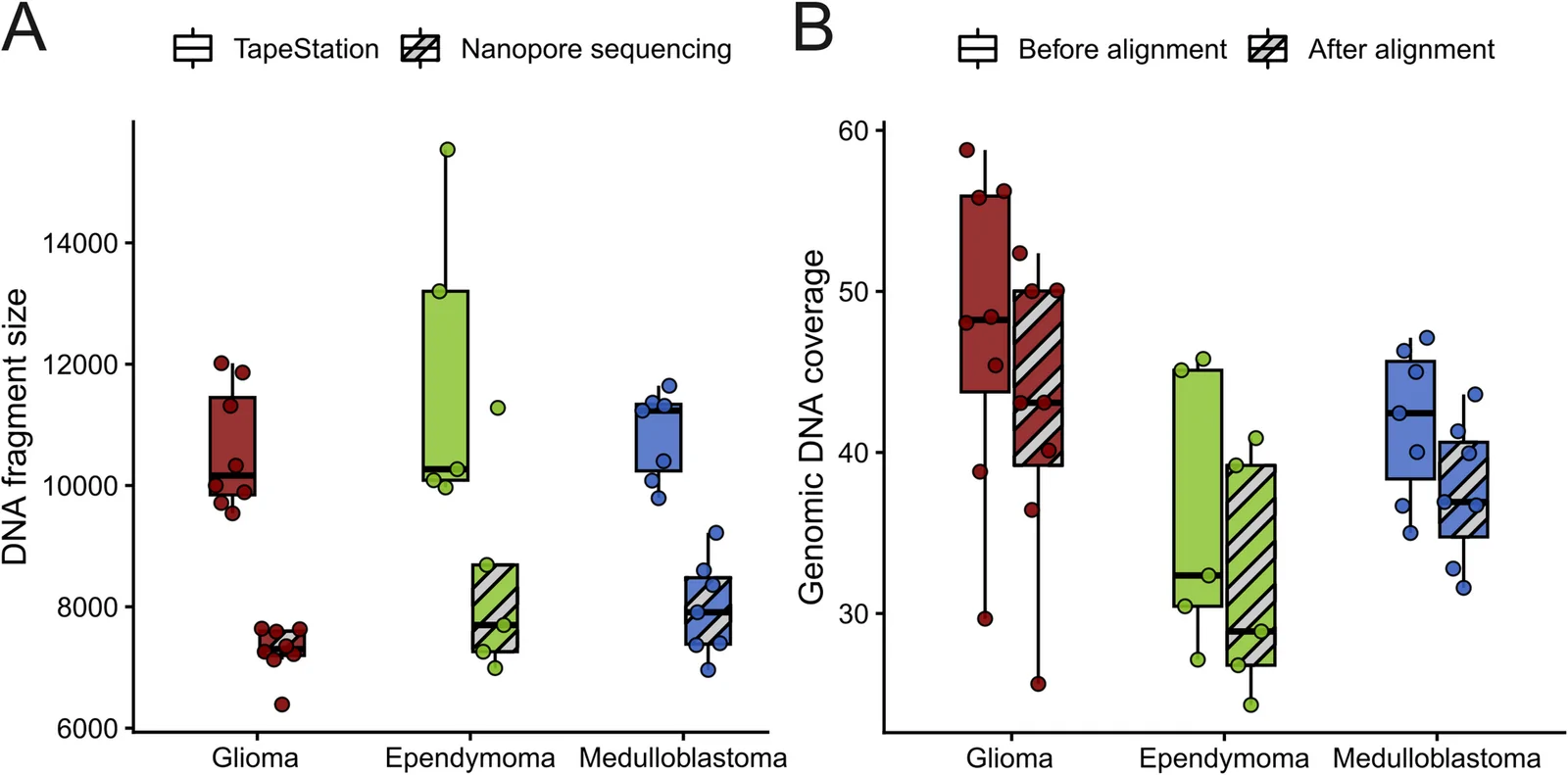
Performance of long-read nanopore sequencing for pediatric CNS tumor characterization. **A** Comparison of DNA fragment sizes measured by TapeStation before library preparation and N50 read lengths obtained from nanopore sequencing. **B** Comparison of genomic DNA coverage before and after alignment to the GRCh38 reference genome. Individual samples are shown as points and grouped according to tumor group

We first evaluated the sequencing performance of long-read nanopore sequencing across pediatric CNS tumors. Nanopore sequencing generated reads with an average N50 read length of 7.79 kb (max: 11.28 kb, min: 6.39 kb). N50 read lengths were consistently shorter than DNA fragment sizes measured with TapeStation prior to library preparation, Fig. 1A. Each sequencing run yielded on average 126 Gb of data (base pairs of DNA sequence) corresponding to a mean genome coverage of approximately 40x per tumor (min: 27x, max: 59x). Genome coverage slightly decreased in all samples following alignment to the human reference genome (GRCh38), Fig. 1B. The degree of variation in aligned coverage differed between tumor types, with the greatest variability observed among diffuse gliomas, followed by ependymomas and medulloblastomas. In contrast, pilocytic astrocytomas showed relatively uniform aligned genome coverage across samples.

### Long-read nanopore sequencing enables integrated whole-genome and haplotype-specific copy-number profiling

**Fig. 2:**
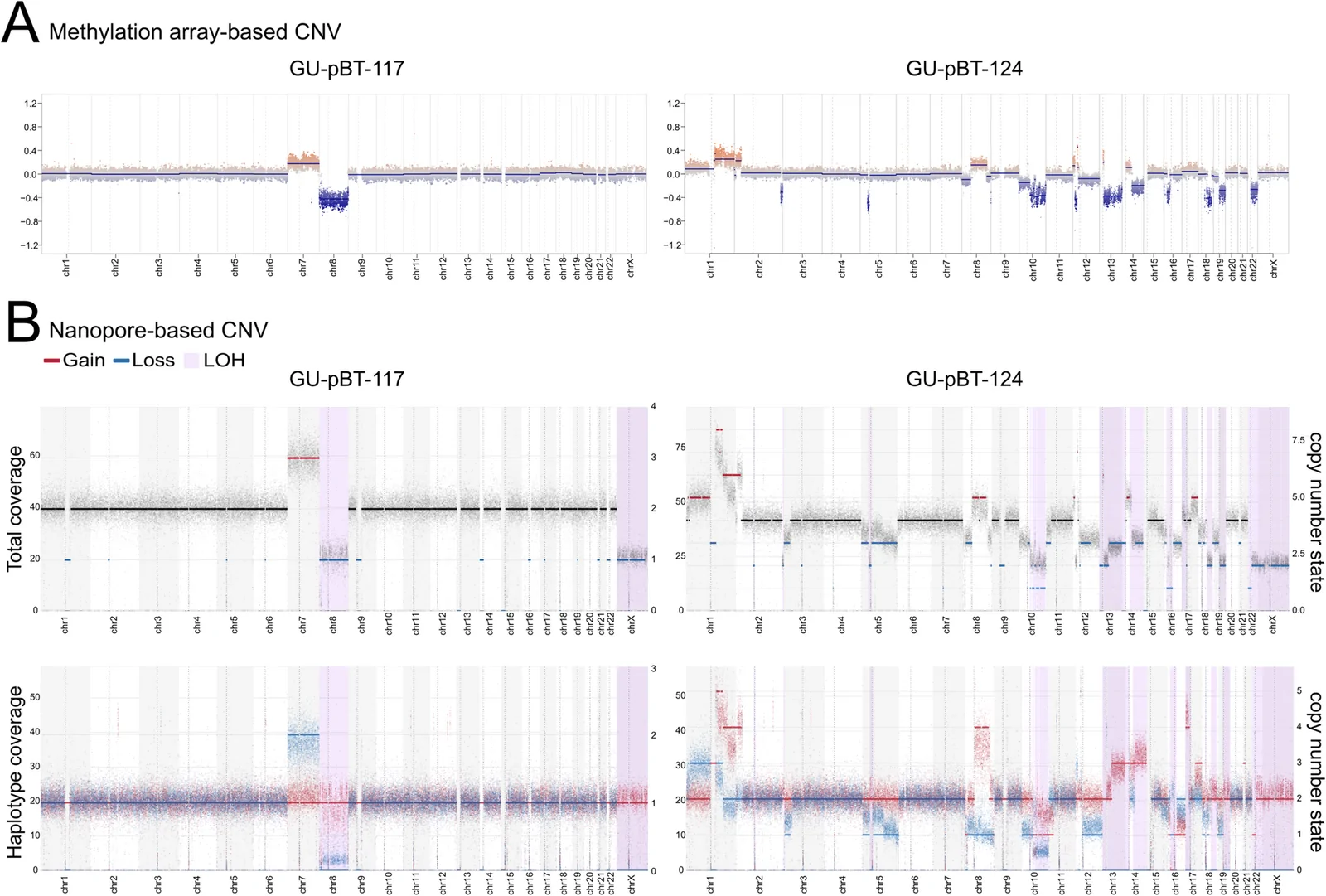
Comparison of copy-number variation (CNV) profiles generated from DNA methylation array and long-read nanopore sequencing. **A** CNV profiles retrieved from methylation array data showing a tumor with a relatively simple copy-number profile (Left; GU-pBT-117) and a highly rearranged tumor (Right; GU-pBT-124). **B** Top panel: CNV profiles inferred from nanopore data using Wakhan. Segment colors indicate copy-number gain (red), copy- number loss (blue) and baseline copy-number (black). Purple shading denotes regions of loss of heterozygosity (LOH). Bottom panel: Haplotype-specific CNV inferred from nanopore data. Red and blue dots represent phased haplotype coverage, horizontal lines indicate inferred haplotype-specific copy-number states, and purple shading indicates LOH regions

Next, we compared CNVs derived from long-read nanopore sequencing with CNVs retrieved from methylation array data. Overall, nanopore-derived CNV profiles showed high concordance with the methylation array–based profiles. Both broad chromosomal alterations and focal copy-number events were consistently detected, demonstrating close agreement between the two approaches. Representative examples of two cases with distinct CNV profiles are shown in Fig. 2A-B.

Quantitative comparison of the matched profiles showed a median Pearson correlation of *r* = 0.816 (IQR 0.683 – 0.935; range 0.363 – 0.989). To assess the specificity of this concordance, each methylation array CNV profile was additionally compared with all 20 nanopore profiles. Matched profiles showed substantially higher correlations than mismatched profiles (median *r* = 0.816 versus 0.111, respectively; paired one-sided Wilcoxon signed-rank test, *P* = 4.8 × 10⁻⁵). The matched nanopore CNV profile was the highest-correlating profile in 18/20 cases (90%) and ranked within the top three in 19/20 (95%) (Supplementary Material 2).

Correlation-based concordance was influenced by the amount of CNVs present within individual profiles. Nanopore CNV signal variability, measured as the SD of the relative CNV signal, was positively associated with matched methylation array–nanopore sequencing Pearson correlation (Spearman ρ = 0.56, *p* = 0.0098, FDR-adjusted *p* = 0.044; Supplementary Material 2 and Supplementary Material 3). A similar association was observed for nanopore CNV signal range (ρ = 0.54, FDR-adjusted *p* = 0.044) (Supplementary Material 3). This effect was particularly apparent in copy-number-flat profiles; GU-pBT-59 had the lowest Pearson correlation (*r* = 0.36) despite a small mean absolute difference between the methylation array and nanopore relative CNV signals (0.034) (Supplementary Material 2).

In addition to conventional copy-number profiling, nanopore sequencing enabled haplotype-specific copy-number through long-read phasing (Fig. 2B). This approach distinguished copy-number changes affecting individual parental haplotypes, allowing visualization of allelic imbalance and LOH that are not directly apparent from copy-number profiles alone.

### Cross-platform evidence for clinically relevant target variants

**Fig. 3:**
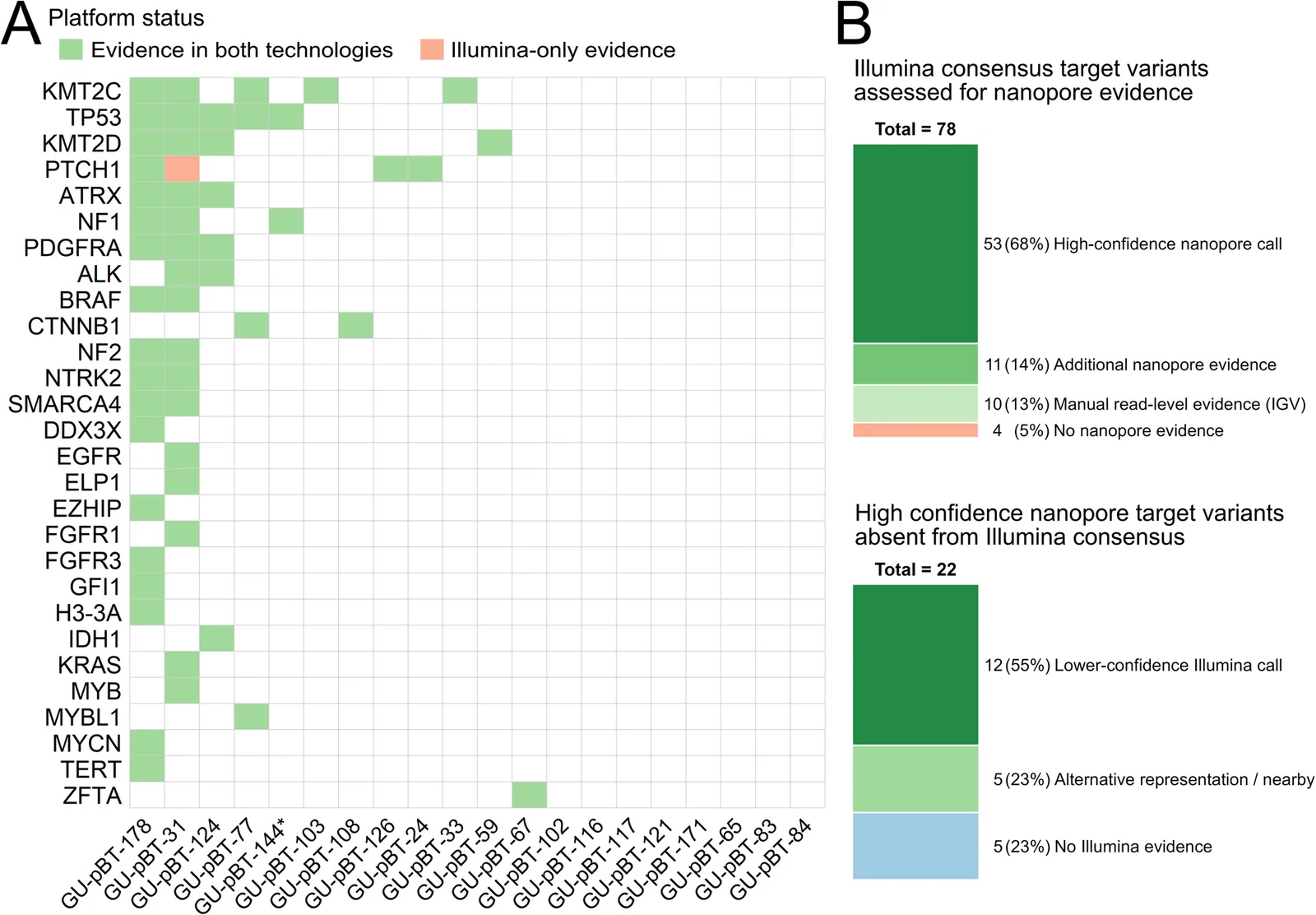
Cross-platform evidence for clinically relevant target variants. **A** Gene-level oncoprint of clinically relevant target-gene alterations across all pediatric samples. Each cell represents a sample–gene combination. Green indicates that at least one target variant in the gene had evidence in both sequencing technologies (Nanopore and Illumina), whereas orange indicates that evidence was restricted to Illumina. Support in both technologies included exact high-confidence calls as well as additional computational or manual evidence identified during review of discordant variants. Individual nanopore-only variants do not result in a nanopore-only cell when another variant in the same sample–gene combination has evidence in both technologies. * = FreeBayes results unavailable. **B** Variant-level evidence summary for clinically relevant target variants. Illumina consensus target variants (top panel) and high-confidence nanopore variants absent from the Illumina consensus (bottom panel) were classified according to the highest level of supporting evidence identified in the opposite sequencing technology, including lower-confidence computational evidence and manual review where applicable. High-confidence nanopore calls correspond to Lumos PASS calls; additional computational nanopore evidence includes variants identified in the complete Lumos callset or Human Variation Workflow. Green shades indicate evidence in both technologies, whereas orange and blue indicate evidence restricted to Illumina and Nanopore, respectively. HVW: Human Variation Workflow. IGV: Integrative Genomics Viewer

Target-gene analysis of Illumina short-read sequencing and long-read nanopore sequencing identified cross-platform evidence for most clinically relevant target variants (Fig. 3A–B, Supplementary Material 4). Among Illumina consensus target variants, 74 of 78 (95%) had nanopore evidence, while 17 of 22 (77%) high-confidence nanopore variants absent from the Illumina consensus had evidence in individual Illumina caller outputs or as alternative/nearby variant representations. Most target variants were concentrated in a subset of samples, particularly the two hypermutated CMMRD cases (GU-pBT-178 and GU-pBT-31).

Among 78 Illumina consensus target variants, 53 (68%) were recovered as exact high-confidence nanopore calls (Lumos PASS) (Fig. 3B). Exact high-confidence nanopore recovery was higher for SNVs (51/68; 75%) than for INDELs (2/10; 20%); however, all 10 INDELs showed nanopore evidence after additional computational or manual review. Interrogation of additional nanopore evidence identified computational support for a further 11 variants (14%), while manual inspection of nanopore alignments in IGV identified read-level support for another 10 variants (13%). Consequently, only four Illumina consensus target variants (5%) remained without detectable nanopore evidence. Three of these (75%) showed low variant allele frequencies (5–12%) despite sequencing depths exceeding 90× in Illumina, whereas the remaining variant (25%) showed a variant allele frequency of approximately 40% with > 100× depth.

Among 22 high-confidence nanopore sequencing target variants absent from the Illumina consensus, 12 (55%) were detected by at least one individual Illumina caller but did not meet the Illumina consensus criterion, and five (23%) corresponded to nearby or alternative variant representations. Only five (23%) high-confidence nanopore target variants lacked detectable Illumina evidence. Four of these (80%) had low nanopore variant allele frequencies (5–7%) at depths of 45–58×, whereas the remaining variant (20%) was supported by a substantially higher nanopore allele frequency (24%) at 58× depth.

Given the low VAF of several discordant target variants, we further assessed SNV recovery across the full Illumina consensus callset. Nanopore sequencing recovery increased markedly with increasing Illumina VAF and reached a high plateau at intermediate-to-high VAFs (Supplementary Material 5). Consistently, samples with higher median Illumina SNV VAF showed higher nanopore SNV recovery (Spearman ρ = 0.56, *p* = 0.0098); this association remained when the two hypermutated CMMRD cases were excluded (ρ = 0.50, *p* = 0.036). In contrast, nanopore SNV recovery was not significantly associated with tumor purity estimated by Wakhan (DNA purity: Spearman ρ = 0.35, *p* = 0.13; cell : ρ = 0.25, *p* = 0.28).

### Structural variant detection and cross-platform breakpoint overlap

**Fig. 4:**
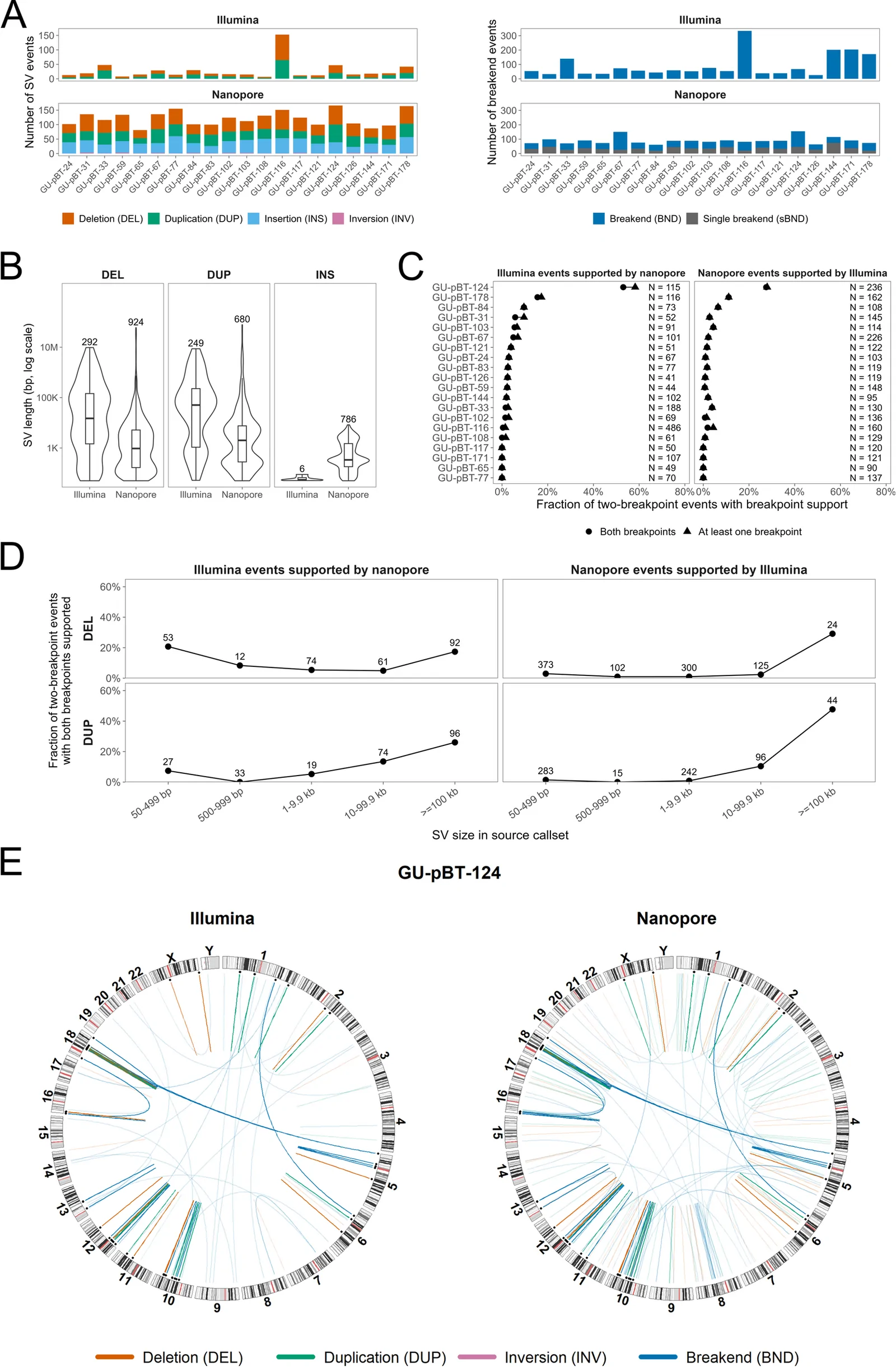
Structural variant profiles and breakpoint overlap between Illumina short-read and long-read nanopore sequencing. **A** Number of PASS structural variant (SV) calls per sample. Classified SVs (DEL, DUP, INS and INV; left) and breakend calls (BND and sBND; right) are shown separately. **B** Size distributions of deletions (DEL), duplications (DUP) and insertions (INS) with defined SV length. Numbers indicate the number of events included in each distribution. **C** Bidirectional, SV-type-independent breakpoint overlap between Illumina and nanopore. For two-breakpoint events from each source dataset, points indicate the fraction for which both breakpoints, or at least one breakpoint, had a corresponding breakpoint on the same chromosome within ± 500 bp in the other dataset. Numbers indicate the total number of evaluable source events per sample. **D** Fraction of deletion and duplication events for which both breakpoints were supported in the other dataset, stratified by SV size in the source dataset. Numbers indicate the number of source events in each size category. **E** Genome-wide SV landscape of GU-pBT-124 for Illumina (left) and nanopore sequencing (right). Links represent two-breakpoint SVs and are colored by SV type. Link opacity indicates breakpoint support in the other dataset (opaque, both breakpoints supported; intermediate, one breakpoint supported; transparent, neither breakpoint supported), and black marks on the chromosome ideograms indicate individual breakpoints supported within ± 500 bp. Cytobands are shown according to GRCh38

SV profiles differed between Illumina short-read and long-read nanopore sequencing (Fig. 4A–B). Across the 20 samples, Nanopore identified 924 deletions, 683 duplications and 786 insertions, compared with 292, 249 and 9, respectively, using Illumina. Conversely, more breakend (BND) events were identified using Illumina (*n* = 1,797) than Nanopore (*n* = 1,085) (Fig. 4A). The size distributions also differed between the two approaches: median deletion and duplication sizes were 15.0 kb and 50.6 kb, respectively, for Illumina, compared with 956 bp and 2.0 kb for Nanopore (Fig. 4B).

Type-independent breakpoint analysis showed low overlap for most sample pairs (Fig. 4C). For Illumina-derived two-breakpoint events, the proportion with both breakpoints supported by Nanopore ranged from 0 to 15.5% in 19 of 20 samples, while GU-pBT-124 showed 53.0% support (61/115 events). In the reciprocal analysis, 0–11.1% of nanopore-derived events had both breakpoints supported by Illumina in 19 of 20 samples, compared with 27.5% (65/236) in GU-pBT-124. Allowing support for only one of the two breakpoints increased overlap only modestly in most samples.

Breakpoint overlap also varied with SV size (Fig. 4D). For events ≥ 100 kb, both breakpoints were supported in the other dataset for 17.4% (16/92) of Illumina deletions and 26.0% (25/96) of Illumina duplications, and for 29.2% (7/24) of nanopore deletions and 47.7% (21/44) of nanopore duplications. A genome-wide representation of GU-pBT-124 illustrates the distribution of shared and dataset-specific SV breakpoints (Fig. 4E).

### Nanopore methylation-based diagnostic classification

**Fig. 5:**
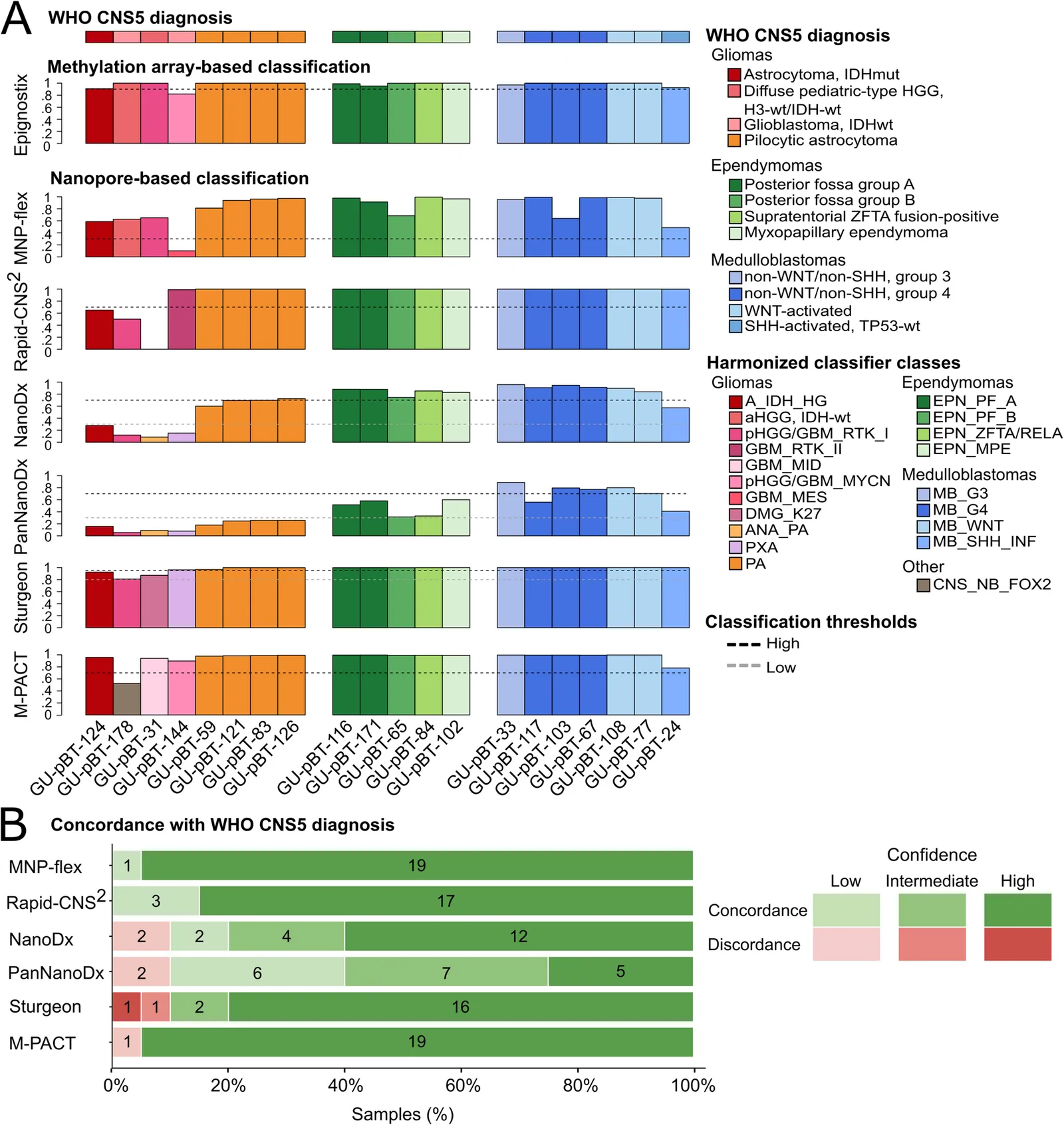
Methylation-based diagnostic classification of pediatric CNS tumors using nanopore-based classifiers. **A** WHO CNS5 diagnoses and methylation classifications according to the Epignostix array-based classifier and six nanopore-based classification pipelines for each pediatric CNS tumor sample. **B** Classifier-specific score/confidence categories and concordance with WHO CNS5 diagnoses. Concordance was assessed independently of classifier score/confidence. Score thresholds and confidence definitions are classifier- and implementation-specific and vary across published studies; the thresholds applied in the current study are described in Materials and methods. Numbers indicate the number of tumors in each category (*n* = 20). Methylation-class assignments were assessed for compatibility with the corresponding WHO CNS5 diagnosis, accounting for differences in nomenclature and classification hierarchy between classifiers. a = adult, p = pediatric

We assessed the utility of nanopore-derived methylation data for molecular classification of pediatric CNS tumors. Methylation-based classifications were compared with the patients’ WHO CNS5 diagnosis using the Epignostix array-based classifier and six nanopore-based methylation classifiers/pipelines, including MNP-Flex, Rapid-CNS^2^, NanoDx, PanNanoDx, Sturgeon and M-PACT (Fig. 5, Supplementary Material 6).

The distribution of classifier scores and confidence categories varied considerably between the nanopore-based classifiers. Using the classifier-specific thresholds, MNP-Flex and M-PACT each yielded 19 of 20 (95%) classifications above their respective cutoffs (≥ 0.30 and ≥ 0.70, respectively), while Rapid-CNS^2^ yielded 17 of 20 (85%) classifications above the applied cutoff (≥ 0.70). Sturgeon yielded 17 of 20 (85%) high-confidence classifications (≥ 0.95), with the remaining three (15%) classified as confident (0.80 –< 0.95). NanoDx yielded 12 of 20 (60%) high-score, 4 of 20 (20%) intermediate-score, and 4 of 20 (20%) low-score classifications, whereas PanNanoDx yielded 5 of 20 (25%) high-score, 7 of 20 (35%) intermediate-score, and 8 of 20 (40%) low-score classifications.

Irrespective of classifier score or confidence, nanopore-based methylation-class assignments showed high concordance with the WHO CNS5 diagnoses. Concordance was observed in 20 of 20 (100%) samples for MNP-Flex and Rapid-CNS^2^, 19 of 20 (95%) for M-PACT, and 18 of 20 (90%) for NanoDx, PanNanoDx, and Sturgeon. Discordant classifications were restricted to three high-grade glioma samples (GU-pBT-178, GU-pBT-31, and GU-pBT-144). Ependymomas, medulloblastomas, pilocytic astrocytomas, and the IDH-mutant astrocytoma (GU-pBT-124) were classified concordantly with their WHO CNS5 diagnoses across all six nanopore classifiers. For GU-pBT-31, diagnosed as diffuse pediatric-type high-grade glioma, H3-wildtype and IDH-wildtype grade 4, MNP-Flex assigned a pediatric high-grade glioma RTK_I methylation class, while Rapid-CNS^2^ and M-PACT assigned the related adult GBM_RTK_I and GBM_MID classes, respectively; these were considered comparable with the WHO CNS5 diagnosis. In contrast, NanoDx and PanNanoDx assigned this sample as ANA_PA and Sturgeon as DMG_K27. For the two patients with the WHO CNS5 diagnoses glioblastoma, IDH-wildtype, GU-pBT-144, was assigned as PXA with NanoDx, PanNanoDx, and Sturgeon and GU-pBT-178 was assigned as CNS_NB_FOXR2 with M-PACT. Comparison with the Epignostix v12.8 array-based methylation reference revealed greater variability at the methylation-class level than when classifications were evaluated against the WHO CNS5 diagnoses. Concordance with Epignostix was observed in 19/20 (95%) samples for MNP-Flex, 18/20 (90%) for M-PACT, and 17/20 (85%) for Rapid-CNS^2^, NanoDx, PanNanoDx and Sturgeon (Supplementary Material 7). Discordant methylation-class assignments were restricted to three diffuse high-grade glioma samples (GU-pBT-31, GU-pBT-144 and GU-pBT-178), including classifications exceeding the applied score thresholds.

The confidence of discordant classifications varied between classifiers. Both discordant NanoDx and discordant PanNanoDx classifications were low-score calls. Of the two discordant Sturgeon classifications, one was classified as confident and one as high confidence, whereas the single discordant M-PACT classification was below the applied cutoff. Thus, classifier score or confidence did not directly correspond to diagnostic agreement.

Wakhan-estimated cell and DNA purity were not significantly associated with classifier scores, the number of classifiers reaching their applied score thresholds, or the number of classifier-assigned methylation classes concordant with the WHO CNS5 diagnosis (all *p* > 0.05; Supplementary Material 8). Notably, discordant classifications were not restricted to low-purity samples; GU-pBT-144 had estimated cell and DNA purities of 1.0 despite discordant classifications by three classifiers.

### Classification performance across sequencing depth

**Fig. 6:**
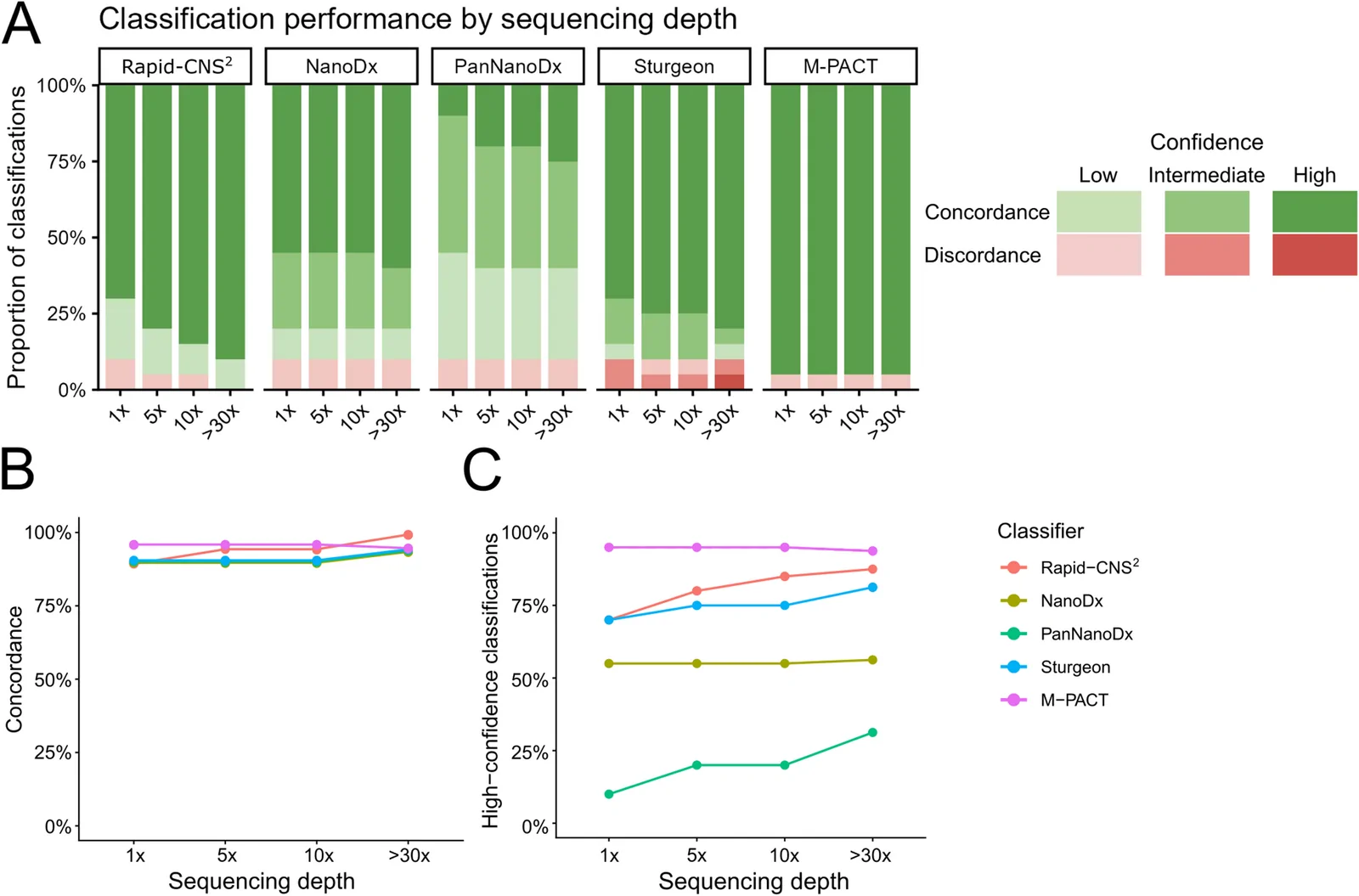
Nanopore methylation-based classification performance across sequencing depths using combined 5mC + 5hmC profiles. **A** Proportions of concordant and discordant classifications across sequencing depths (1×, 5×, 10×, and > 30×) for five nanopore-based methylation classifiers. Classifications are stratified by classifier-specific confidence categories (low, intermediate, and high). Green shades indicate classifications concordant with the integrated WHO CNS5 diagnosis, whereas red shades indicate discordant classifications. **B** Proportion of classifications concordant with the WHO CNS5 diagnosis, irrespective of classification confidence, across sequencing depths. **C** Proportion of classifications reaching the classifier-specific high-confidence threshold across sequencing depths. Confidence categories and thresholds were defined separately for each classifier as described in Materials and methods. Each sequencing-depth group included 20 tumor samples

To evaluate the effects of long-read nanopore sequencing depth on methylation-based classification, we compared classifications at 1×, 5×, 10×, and > 30× coverage using combined 5mC + 5hmC profiles (Fig. 6). Concordance with the WHO CNS5 diagnosis remained largely stable across sequencing depths, ranging from 90 to 100% across classifiers and depths (Fig. 6A–B). In contrast, the proportion of classifications reaching classifier-specific high-confidence thresholds varied more substantially. M-PACT maintained high-confidence classifications across all sequencing depths, whereas Rapid-CNS^2^ and Sturgeon showed increasing proportions of high-confidence classifications with increasing coverage. NanoDx showed relatively stable high-confidence classification rates, while PanNanoDx showed the lowest proportions of high-confidence classifications, although these increased with sequencing depth (Fig. 6A, C).

The corresponding analysis using 5mC-only profiles showed similar overall patterns, with diagnostic concordance largely maintained across sequencing depths (Supplementary Material 9). However, the proportion of high-confidence classifications was generally lower than with combined 5mC + 5hmC profiles for Rapid-CNS^2^, NanoDx, and PanNanoDx, whereas differences were smaller for Sturgeon and M-PACT. These findings indicate that the effect of modified-base representation on classification confidence varies between classifiers.

Based on the sequencing yield of the full nanopore sequencing runs, the reduced-depth datasets corresponded to estimated sequencing times of approximately 1.8 h for 1×, 9 h for 5×, and 18 h for 10× coverage. As downstream methylation processing and classification required substantially less time than sequencing, these depths correspond to approximate times to methylation classification results of 2 h, 9–10 h, and 19 h, respectively. These estimates assume approximately proportional accumulation of sequencing coverage and therefore represent projected rather than prospectively measured turnaround times.

For comprehensive genomic analysis at approximately 40× coverage, sequencing required approximately 72 h. The complete Lumos analysis pipeline required approximately 8.1 h wall time, corresponding to an estimated total time of approximately 3–4 days from sequencing initiation to comprehensive molecular results.

## Discussion

Comprehensive molecular characterization has become essential for the diagnosis and clinical management of children with CNS tumors, yet current diagnostic workflows typically rely on multiple complementary assays including short-read sequencing and methylation array profiling. In this study we evaluated the performance and applicability of long-read nanopore sequencing as an integrated approach for pediatric CNS tumor diagnostics. Our findings should be interpreted in the context of the relatively small, retrospective cohort and the selected range of CNS tumor groups included, which limit their generalizability across the broader spectrum of pediatric CNS tumors, particularly rare molecular entities. Nevertheless, this study provides a foundation for further evaluation in larger, more diverse prospective cohorts. Robust sequencing performance is a prerequisite for reliable downstream molecular analyses. In this study, nanopore sequencing consistently generated sufficient read lengths and genome coverage across all pediatric CNS tumor groups to support variant detection, copy-number profiling, SV analysis, and methylation-based classification. Although aligned genome coverage varied between tumor groups, the observed sequencing metrics were sufficient for successful molecular characterization across the study cohort, supporting the feasibility of nanopore sequencing in routine diagnostic workflows.

Our results demonstrated that nanopore sequencing reliably recapitulated the copy-number landscape of pediatric CNS tumors, reproducing both broad chromosomal alterations and focal copy-number events identified by DNA methylation array analysis. This was supported by the quantitative comparison of matched CNV profiles, which showed a median cross-platform Pearson correlation of 0.82. Moreover, in 18 of 20 cases, the matched nanopore profile showed the highest correlation with its corresponding Illumina profile among all nanopore profiles, supporting patient-specific concordance between the two approaches.

Differences between the platforms should, however, be interpreted in the context of their distinct measurement principles and genomic resolution. Methylation array-derived CNV profiles are inferred from probe intensities, whereas nanopore-derived profiles are based on sequencing coverage and may resolve smaller copy-number alterations not captured by the methylation array. Our analysis was therefore restricted to the quantitative comparison with genomic loci interrogated by the methylation array. Cross-platform correlation was also influenced by the extent of variation within individual CNV profiles, with greater nanopore CNV signal variability associated with higher methylation array–nanopore Pearson correlation. This supports the interpretation that copy-number-flat profiles provide limited dynamic range for correlation-based comparisons. Cross-platform correlation was associated with the extent of CNV signal variation within individual profiles, with greater nanopore CNV signal variability associated with higher methylation array–nanopore Pearson correlation. This suggests that the limited dynamic range of flat CNV profiles can contribute to lower correlation-based concordance, although CNV signal variability did not fully account for the differences between individual profiles. GU-pBT-59, which had the lowest Pearson correlation, showed a particularly flat CNV profile despite a small mean absolute difference between the relative CNV signals. In addition, nanopore sequencing enabled haplotype-resolved copy-number analysis through long-read phasing, providing information beyond that available from conventional array-based CNV analysis. This enabled resolution of haplotype-specific gains and losses, as well as regions of LOH, thereby providing additional insight into the allelic and structural complexity within the tumor genome.

The comparison of clinically relevant target variants demonstrated that restricting analyses to high-confidence PASS calls did not capture the full extent of cross-platform sequencing evidence. Many variants initially classified as platform-specific showed evidence in the other technology following additional computational interrogation or manual read-level inspection. This was particularly evident for INDELs, for which exact high-confidence nanopore call recovery was lower than for SNVs, although all Illumina consensus INDELs showed nanopore evidence following extended review. Importantly, this retrospective evidence was considered separately from primary pipeline detection. These findings suggest that a considerable proportion of apparent discordance reflects differences in variant representation, caller behavior and filtering strategies rather than genuine absence of sequencing signal.

Part of this discordance is also likely attributable to differences in the analytical frameworks applied to the two datasets. Whereas the Illumina reference set was derived from tumor–normal sequencing, nanopore variants were called using a tumor-only workflow. The observation that many clinically relevant variants were recovered only through additional computational interrogation suggests that the high-confidence filtering strategy used here is conservative for somatic target variant detection.

Variant allele frequency was associated with cross-platform variant recovery, with lower nanopore recovery of Illumina consensus SNVs at low VAF. A similar pattern was observed among the remaining discordant clinically relevant variants: most variants detected exclusively by either technology had low VAFs, suggesting that low-frequency variants represent a general challenge for cross-platform concordance rather than a limitation unique to nanopore. Importantly, the relationship between VAF and nanopore recovery was gradual and did not support a discrete detection threshold. Moreover, occasional discordance at higher VAF indicates that additional factors, including variant filtering, representation and read mapping, also contribute to differences between platforms. Although lower VAF was associated with reduced nanopore SNV recovery, no corresponding association was observed between SNV recovery and tumor purity estimates, suggesting that sample purity alone did not explain the observed differences in variant recovery.

The Neurofibromatosis type I gene *(NF1)* remained a notable exception. Manual inspection of nanopore sequencing alignments showed that most discordant *NF1* variants were visible at the read level but were predominantly supported by reads with mapping qualities close to zero. This suggests that reduced recovery in *NF1* primarily reflects challenges in long-read alignment rather than complete absence of sequencing signal or limitations of variant calling itself. Improved long-read mapping algorithms may therefore substantially improve nanopore performance in this locus.

Overall, these findings indicate that apparent discordance between Illumina and nanopore target variants can arise from bioinformatic processing as well as differences in sequencing signal, highlighting bioinformatic optimization as an important avenue for improving clinically relevant variant recovery from long-read sequencing.

Structural variant profiles differed in both SV type and size distributions between Illumina short-read and nanopore sequencing, with lower cross-platform overlap than observed for small variants. In 19 of 20 samples, complete two-breakpoint support ranged from 0 to 15.5% for Illumina-derived events and 0–11.1% for nanopore-derived events, while GU-pBT-124 was a notable exception (53.0% and 27.5%, respectively). Low overlap between short- and long-read SV calls has also been reported by McAuley et al., who found direct SV concordance of 2.4% and 0.3% in pre- and post-treatment tumor samples from a patient with rectal cancer sequenced using both Illumina and nanopore [11]. Although their concordance definition differs from our breakpoint-based analysis, their findings highlight the challenges of direct comparison between short- and long-read SV callsets.

In our study, differences in sequencing and SV-calling approaches may therefore contribute to the observed discordance. In addition, Illumina and nanopore sequencing were performed on different DNA aliquots, and spatial intratumor heterogeneity may contribute to differences between the SV profiles. Supporting this possibility, Xie et al. reported spatial SV heterogeneity in glioblastoma-derived stem cell lines established from central and peripheral regions of the same tumor (Jaccard similarity 0.42) [23]. The relative contributions of technical and spatial differences cannot be resolved with the present study design.

A principal aim of this study was to evaluate the performance of different nanopore methylation-based classifiers for diagnostic classification of pediatric CNS tumors. Overall, methylation classifications showed high agreement with the integrated WHO CNS5 diagnoses, with 90–100% concordance across the evaluated classifiers. Ependymomas, medulloblastomas, and pilocytic astrocytomas showed concordant classifications across all evaluated classifiers, although not all predictions reached the respective classifier-specific confidence thresholds. This suggests that, within our cohort, the methylation profiles of these tumor types were sufficiently distinct to be reproducibly recognized across different classification algorithms. In contrast, greater variability was observed for diffuse gliomas. While the IDH-mutant astrocytoma (GU-pBT-124) was concordantly classified by all the classifiers, both CMMRD-associated gliomas (GU-pBT-178 and GU-pBT-31) showed greater variability in assigned methylation classes. This likely reflects not only the biological and epigenetic heterogeneity of pediatric diffuse gliomas [5, 21], which remains a challenge for molecular classification irrespective of analytical platform, but also the limited representation of rare molecular subtypes, such as CMMRD-associated gliomas, in the reference datasets used to train current methylation classifiers. Methylation subclasses, particularly within glioblastoma, may represent less clearly delineated and potentially overlapping biological states, and intratumor epigenetic heterogeneity may further contribute to variability in subclass assignment. We have previously demonstrated that spatially separated biopsies from the same glioblastoma could receive different methylation subclass assignments despite consistent classification at the broader diagnostic level [20]. Differences in training datasets, reference cohorts and machine-learning approaches used to develop the individual classifiers may further contribute to divergent subclassification for tumors with atypical or similar methylation profiles.

The proportion of classifications exceeding classifier-specific classification thresholds varied substantially across the nanopore pipelines. Importantly, the proportion of samples exceeding these thresholds did not directly reflect concordance with WHO CNS5 diagnoses. Irrespective of classifier score, nanopore methylation-based classifications were concordant with WHO CNS5 diagnoses in 100% for MNP-Flex and Rapid-CNS^2^, 95% for M-PACT, and 90% for NanoDx, PanNanoDx, and Sturgeon. Methylation-class agreement with the methylation array-based Epignostix classifier was also high but varied between classifiers, reflecting differences in classifier nomenclature and the resolution of the methylation classes represented in their respective reference frameworks. The observed differences between classifiers regarding classification rates likely reflect their distinct approaches to sparse nanopore methylation data. Sturgeon and M-PACT consistently achieved high-confidence classifications, likely owing to architectures optimized for sparse methylomes. Rapid-CNS^2^ and NanoDx use different modelling strategies, which may contribute to differences in classification performance, particularly for diffuse gliomas. In contrast, PanNanoDx showed the lowest classification confidence, which may reflect reduced sensitivity for CNS tumor subclassification due to its broader pan-cancer design. Together, these findings highlight the trade-offs between classification confidence, computational speed, and model scope when selecting nanopore-based methylation classifiers. Our findings further illustrate that classifier confidence scores should not be interpreted as direct measures of diagnostic accuracy, as some below-threshold classifications remained concordant with WHO CNS5 diagnoses, whereas discordant classifications occasionally occurred despite high confidence scores. Furthermore, tumor purity did not appear to account for the observed variation in methylation classification, as neither estimated cell nor DNA purity was associated with classifier scores, threshold attainment, or concordance with the WHO CNS5 diagnosis.

The sequencing-depth analysis further showed that diagnostic concordance and classifier confidence responded differently to reduced methylation data availability. Concordance with the WHO CNS5 diagnosis was largely maintained across sequencing depths, whereas the proportion of classifications reaching classifier-specific confidence thresholds generally increased with sequencing depth, although the magnitude of this improvement varied between classifiers and sequencing depths. M-PACT and Sturgeon maintained relatively high classification scores across the evaluated sequencing depths, whereas Rapid-CNS^2^, NanoDx, and particularly PanNanoDx, showed greater improvements in score or confidence category with increasing depth. These findings are consistent with differences in how the classifiers were designed to process sparse methylation data and indicate that increasing sequencing depth does not affect all classification approaches uniformly. This is relevant for diagnostic implementation, as lower sequencing depth could reduce sequencing time and data requirements, but the depth required to maintain classification performance may depend on the classifier used. Importantly, the present downsampling analysis evaluates computational performance at reduced coverage rather than turnaround time in a prospective diagnostic workflow and therefore does not establish a minimum sequencing depth for clinical use.

Comparison of the combined 5mC + 5hmC profiles with the corresponding 5mC-only profiles showed that inclusion of 5hmC generally resulted in similar or higher classification scores, although the magnitude of the difference varied among classifiers. The limited differences observed for M-PACT and Sturgeon suggest that inclusion of 5hmC had relatively little effect on classification performance for these methods under the conditions evaluated. Combining 5mC and 5hmC also facilitates comparison with methylation-array-derived reference data, which do not distinguish between these two cytosine modifications. However, the effect was not uniform across classifiers, supporting the use of classifier-specific preprocessing rather than assuming that a single methylation representation is optimal for all methods.

Turnaround time is an important consideration for potential clinical implementation. Although the sequencing runs in this study were performed for 72 h and generated approximately 40× genome coverage, the sequencing-depth analysis indicates that methylation-based classification may be possible substantially earlier. Based on the observed sequencing yield, 1×, 5×, and 10× coverage would be expected after approximately 1.8, 9, and 18 h of sequencing, corresponding to estimated methylation-classification results after approximately 2, 9–10, and 19 h, respectively. In contrast, comprehensive analysis at approximately 40× coverage was estimated to require approximately 3–4 days from sequencing initiation, including approximately 8 h for the full Lumos workflow. These estimates should not be interpreted as prospectively measured clinical turnaround times, because the reduced-depth datasets were generated retrospectively and the sequencing-rate estimates assume approximately proportional accumulation of coverage. Prospective studies using shortened sequencing runs and real-time data processing are required to establish achievable clinical turnaround times.

An additional challenge for clinical implementation is the interpretation and comparability of classifier scores. Classification thresholds are not necessarily transferable between different implementations of the same classifier. For example, the originally validated Rapid-CNS^2^ classifier used a score threshold of 0.30, whereas the random forest implementation incorporated in ROBIN was adapted to classification from sparse genomic coverage and uses a different scoring framework and threshold [3, 13]. Similar variation in score interpretation is apparent across implementations of other classifiers, including NanoDx. Thus, classifier scores and thresholds should be interpreted in the context of the specific model, training strategy, and sequencing conditions for which they were developed. Systematic benchmarking, calibration, and validation of these thresholds will therefore be required before nanopore-based methylation classifiers can be implemented as standardized clinical diagnostic tools.

## Conclusion

Our study demonstrates that nanopore long-read sequencing analysis enables integrated molecular characterization of pediatric CNS tumors. Nanopore sequencing provides multiple layers of clinically relevant information on sequence variants, SVs, CNVs and DNA methylation-based classification. Cross-platform comparisons showed a median correlation of *r* = 0.816 for copy-number profiles and nanopore evidence for 95% (74/78) of clinically relevant small variants detected by Illumina, while SV analysis showed limited breakpoint overlap between the two sequencing approaches. Despite differences in classifier-specific score distributions, methylation-class assignments showed high concordance with WHO CNS5 diagnoses across all evaluated nanopore-based classifiers. Although challenges remain for heterogeneous tumors such as diffuse gliomas, our findings support the feasibility of integrating multiple diagnostic modalities into a single nanopore-based workflow. Prospective studies in larger, diagnostically diverse cohorts will be important to further establish role of nanopore long-read sequencing in routine clinical practice.

Additional Files.

## Supplementary Information

Below is the link to the electronic supplementary material.

Supplementary Material 1

Supplementary Material 2

Supplementary Material 3

Supplementary Material 4

Supplementary Material 5

Supplementary Material 6

Supplementary Material 7

Supplementary Material 8

Supplementary Material 9

Supplementary Material 10

## Data Availability

Illumina short-read sequencing data and DNA methylation array data was obtained from The Swedish Childhood Tumor Biobank. Data generated in this study are available from the corresponding author upon reasonable request, subject to applicable legal and ethical restrictions and a data sharing agreement.

## Abbreviations

CMMRD: Constitutional mismatch repair deficiency
CNS: Central nervous system
CNV: Copy-number variation
FF: Fresh-frozen
FFPE: Formalin-fixed Paraffin-embedded
HVW: Human variation workflow
IDH: Isocitrate dehydrogenase
IGV: Integrative genomics viewer
LOH: Loss of heterozygosity
ONT: Oxford nanopore technologies
SNV: Single nucleotide variant
SV: Structural variant
WHO: World Health Organization
