# Supplementary material for: Diagnostic evaluation of long-read nanopore sequencing for pediatric CNS tumors
Source: Acta Neuropathol Commun. 2026 Oct 27;14:224. doi: 10.1186/s40478-026-02466-w (PMC13711757; doi:10.1186/s40478-026-02466-w)
Supplement: Supplementary file 1 [file 40478_2026_2466_MOESM10_ESM.docx]

## Supplementary Material

**Supplementary Material 1.** Software versions and key computational parameters used for data processing and analysis. Parameters shown represent key non-default or analysis-defining settings.

**Supplementary Material 2.** Quantitative concordance of copy-number variation profiles derived from methylation arrays and nanopore sequencing. Cross-platform CNV concordance was evaluated across 20 matched tumors. The table reports the number of methylation array CNV segments and patient-level Pearson correlation, Spearman correlation, cosine similarity, and mean absolute difference between methylation array and nanopore relative CNV signals. Matched profiles were additionally compared with mismatched nanopore CNV profiles to assess patient-specific concordance and determine the rank of each matched profile. CNV signal variability was quantified using the standard deviation (SD), interquartile range (IQR), and range of segment-level relative CNV signals for both platforms.

**Supplementary Material 3.** Association between nanopore copy-number variation signal variability and cross-platform CNV concordance. Scatter plot showing the relationship between nanopore copy-number variation (CNV) signal variability, quantified as the standard deviation (SD) of the segment-level relative CNV signal, and matched methylation array–nanopore CNV concordance, quantified using Pearson correlation (r), across 20 tumors. Each point represents one tumor. The blue line indicates the fitted linear trend and the shaded area represents the 95% confidence interval. The association was assessed using Spearman rank correlation (ρ = 0.56, p = 0.0098; Benjamini–Hochberg FDR-adjusted p = 0.044).

**Supplementary Material 4.** Variant-level cross-platform evidence for clinically relevant target-gene variants. Variant-level evidence for Illumina consensus and high-confidence nanopore target variants, including caller-specific VAF and sequencing depth were available. For Illumina callers, VAF and depth are reported only for exact variant matches; values are left blank for alternative or nearby variant representations or when no numeric value was available in the caller output. Cross-platform evidence indicates whether support was identified by primary variant calling, additional computational interrogation, or manual read-level inspection.

**Supplementary Material 5.** Association between variant allele frequency and nanopore SNV recovery. A) Pooled recovery of Illumina consensus SNVs by nanopore sequencing across the cohort according to Illumina variant allele frequency (VAF). B) Per-sample nanopore SNV recovery in relation to median Illumina SNV VAF. CMMRD cases are indicated by triangles. Spearman correlation is shown for all samples.

**Supplementary Material 6.** Methylation-based classifications and tumor purity estimates

**Supplementary Material 7.** Concordance of nanopore-based methylation classifications with the Epignostix v12.8 reference classification. Horizontal stacked bars show the proportion of samples (n = 20) with methylation-class assignments concordant (green) or discordant (red) with the harmonized Epignostix v12.8 array-based methylation classification. Colour intensity represents harmonized classifier-specific score/confidence categories (low, intermediate, and high), with darker shades indicating higher scores or confidence. Numbers within bar segments indicate the number of samples in each category. Concordance was assessed independently of classifier score or confidence. Classifier-specific score thresholds and confidence definitions differ between methods and implementations and are detailed in the Methods and Supplementary Data.

**Supplementary Material 8.** Concordance, classifier confidence, discordant classifications, and tumor-purity analyses for nanopore methylation-based CNS tumor classification. Concordance was assessed between nanopore-based methylation classifications and the Epignostix v12.8 array-based reference classification, irrespective of classifier score or confidence category. The file summarizes classifier-specific concordance and confidence distributions, details discordant classifications and their corresponding scores/confidence categories, and provides sample-level classifications and concordance assessments. Associations between Wakhan-estimated cell and DNA purity and classifier scores, the number of WHO CNS5-concordant classifiers, and the number of classifiers reaching their applied score/confidence thresholds are also reported.

**Supplementary Material 9.** Methylation-based classification performance across sequencing depths using 5mC-only profiles. A) Proportions of concordant and discordant classifications across sequencing depths (1×, 5×, 10×, and >30×) for five nanopore-based methylation classifiers using 5mC-only profiles. Classifications are stratified by classifier-specific confidence categories (low, intermediate, and high). Green shades indicate classifications concordant with the integrated WHO CNS5 diagnosis, whereas red shades indicate discordant classifications. B) Proportion of classifications concordant with the WHO CNS5 diagnosis, irrespective of classification confidence, across sequencing depths. C) Proportion of classifications reaching the classifier-specific high-confidence threshold across sequencing depths. Dashed lines indicate results obtained using 5mC-only profiles. Confidence categories and thresholds were defined separately for each classifier as described in the Methods. Each sequencing-depth group included 20 tumor samples.
